# Biomarkers for Diagnosis of Axial Spondyloarthritis, Disease Activity, Prognosis, and Prediction of Response to Therapy

**DOI:** 10.3389/fimmu.2019.00305

**Published:** 2019-03-07

**Authors:** Walter P. Maksymowych

**Affiliations:** Department of Medicine, University of Alberta, Edmonton, AB, Canada

**Keywords:** biomarker, axial spondyloarthritis, ankylosing spondylitis, diagnosis, prognosis, imaging, multiomics

## Abstract

There exists a major unmet need for biomarkers that can identify axial spondyloarthritis (axSpA) early after disease onset because of the availability of highly effective therapies. Several recent reports have examined the autoantibody response in patients with axSpA through the use of protein microarrays and protein-protein interactions although diagnostic performance of biomarkers identified to date has been inadequate. An example of such a biomarker is protein phosphatase magnesium-dependent 1A. Antibodies to the human leukocyte antigen class II-associated invariant chain peptide (anti-CD74) are candidate diagnostic biomarkers but sensitivity declines with increasing duration of disease. Metabolomic studies have employed nuclear magnetic resonance (NMR) spectrometry to identify disease-specific metabolites related to fat metabolism and intestinal microbial metabolism. A second major unmet need exists for biomarkers of disease activity that have superiority over standard C-reactive protein assessment and reflect MRI inflammation in the axial spine. Several biomarkers reflecting inflammation (calprotectin), angiogenesis (vasoactive endothelial growth factor), and connective tissue turnover (C2M, C3M, and citrullinated metalloproteinase degraded fragment of vimentin) have recently been shown to reflect disease activity when compared with clinical outcomes but comparisons with MRI inflammation are very limited. With increasing availability of highly effective but costly therapies, a third unmet need is biomarkers that can predict response to therapies with different mechanisms of action and are superior to C-reactive protein. Calprotectin is currently the only candidate. Although there are as yet no proven therapies for preventing progression of disease there is an unmet need for biomarkers of prognosis that are more responsive than radiography. Aside from CRP no consistent candidates have emerged. Future studies will need to be prospective, include consecutive patients presenting with undiagnosed back pain, and use more reliable and objective endpoints such as MRI inflammation. Moreover, it has become evident that targeted biomarker studies have not been successful in identifying clinically useful biomarkers and technologies that can simultaneously assess “multiomic” markers will need to be analyzed for future advances. These include more sophisticated metabolomic profiling and universal metabolome-standard (UMS) methodology, next generation RNA sequencing, and affinity-based quantitative proteomics based on the use of nucleic acid binders such as the aptamer-based SOMAscan assay.

## Introduction

Axial spondyloarthritis (axSpA) is a disorder that leads to inflammation in the sacroiliac joints and spine, large peripheral joints, specific entheses, and extra-articular structures such as the anterior uvea and aorta. It is associated with formation of new bone in the axial skeleton that leads to intervertebral and facet joint ankylosis and functional disability. New therapeutic advances have a major impact on inflammation thereby leading to alleviation of signs and symptoms of disease but major challenges remain. Onset of disease is typically in the third and fourth decades of life with lower back and buttock symptoms that also occur frequently in non-specific back disorders. Consequently, diagnosis is often delayed by 5–10 years. Early diagnosis and institution of appropriate anti-inflammatory therapy results in higher rates of clinical remission but early diagnosis is dependent on access to magnetic resonance imaging (MRI). Consequently, there is an unmet need for biomarkers of diagnosis. Once treatment has been instituted it is necessary to monitor its impact. This is presently largely confined to self-reported measures gauging the severity of back symptoms and the relatively insensitive serological marker, C-reactive protein (CRP). Serial assessment of MRI scans for objective evaluation of inflammation is not feasible. Consequently, there is an unmet need for biomarkers of disease activity, which reflect MRI inflammation and which can facilitate appropriate titration of treatment. Early institution of therapy could also preclude the development of structural lesions such as ankylosis but this would require the ability to predict disease outcome with a high degree of probability. In addition, it is important to identify patients at risk of developing serious comorbidities associated with this disease, such as inflammatory bowel disease. Such prediction models do not currently exist and so there is an unmet need for biomarkers reflecting prognostic risk. Recent clinical trials have demonstrated the efficacy of several agents with different mechanisms of action but choosing appropriate treatment amongst an increasing array of options remains empiric (1–3). Consequently, there is an unmet need for biomarkers that can help personalize treatment options. This literature review aims to assess recent advances in the field in the context of these unmet needs.

## Diagnostic Biomarkers

Most studies performed to date have assessed biomarkers in small cross-sectional studies of patients with well-established disease defined according to the modified New York classification criteria for ankylosing spondylitis (4). This is appropriate for initial identification of biomarkers with diagnostic potential but subsequent studies should test the performance of biomarkers in appropriate diagnostic settings i.e., the patient presenting to the rheumatologist with undiagnosed back pain. Because clinical assessment alone may lack diagnostic certainty, diagnostic evaluation in such settings should include both MRI and prospective evaluation over at least 2 years to allow for the evolution of features characteristic of disease. Radiographic assessment of sacroiliac joints often lacks sufficient reliability in early disease. Diagnostic performance should be sufficient to be considered of clinical utility for individual patients supported by metrics such as a positive predictive value (PPV) of >80% and positive likelihood ratio (LR) of >5. Conversely, because referral for back pain is so common in the age group that develops axSpA it is important that such a biomarker can effectively rule out disease with performance metrics such as >80% negative predictive value (NPV) and negative LR of <0.2. The International Soluble Biomarker Working Group for the Outcome Measures in Rheumatology Consortium has developed additional performance characteristics that can be considered essential for a diagnostic biomarker such as stability of the biomarker at room temperature and after prolonged periods of storage, feasibility of assay use and well-defined standards, and limited variability due to age, gender, diurnal factors, body mass index, ethnicity, and disease duration (5). The recent literature has demonstrated a gradual shift from studies of candidate biomarkers derived from knowledge of pathogenesis toward multiomic non-hypothesis driven identification of panels of candidate biomarkers. But validation studies focused on the criteria described in the foregoing text remain very limited in axSpA. This review aims to describe recent work done in the field toward identification of biomarkers relevant to axSpA and to interpret the findings in the context of target performance measures and recommended study designs. The recent literature is summarized in the Table 1.

**Table 1:** Summary of recent literature evaluating biomarkers for specific unmet needs in axSpA.

**Biomarker**	**Description**	**Role in AxSpA**	**Primary studies**
Anti-CD74	Antibody to extracellular HLA-class II γ-chain	Diagnosis	56% axSpA, 4% controls (6); 69% axSpA, 45% PsA, 11% RA, 15% SLE, 0.8% controls (6); 85.1% axSpA, 7.8% controls (7); 47% axSpA, 4.7% controls (IgA) (8); 46.4% axSpA, 47.9% controls (IgG) (9)
Anti-PPM1A	Antibody to protein phosphatase magnesium-dependent 1A, a Ser/Thr protein phosphatase that regulates BMP and Wnt signaling	Diagnosis	66.7% of axSpA, 26.7% controls (10)
Micro-RNAs	Composite signature based on miR-146a-5p, miR-125a-5p, miR-151a-3p, miR-22-3p, miR-150-5p, miR-451a	Diagnosis	90.6% axSpA, 7.5% controls (11)
Calprotectin	Heterodimer of S100A8 and S100A9	Disease activity	Serum and fecal levels correlated with BASDAI, ASDAS, CRP, and microscopic bowel inflammation (12–15)
Interleukin 6	Cytokine non-specifically elevated in axSpA	Disease Activity	Associated with CRP and MRI inflammation (16)
Hepatocyte growth factor	Secreted by mesenchymal cells, suppresses inflammation and enhances osteoblastic differentiation of mesenchymal cells	Disease Activity	Associated with acute phase reactants (17, 18)
C1M, C6M, VICM	Degradation fragments of types I (C1M), and VI (C6M) collagens and vimentin generated by matrix metalloproteinase (MMP)	Disease Activity	Associated with CRP (19)
Micro-RNAs	MiR-146a, miR-155 downregulate toll-like receptor signaling	Disease Activity	Associated with CRP, BASDAI and pro-inflammatory cytokines (11, 20)
CRP	C-reactive protein, acute phase reactant	Prognosis	The only consistent predictive biomarker of disease progression (21–26)
MIF	Macrophage inhibitoryfactor, a mediator directly enhancing osteogenesis	Prognosis	Higher levels in patients with progression of radiographic changes in the spine (27)
Visfatin	Adipokine directly involved in osteogenesis	Prognosis	Predicts radiographic progression independently of CRP (28)
Micro-RNAs	Composite signature based on miR-125a-5p, miR-151a-3p, miR-150–5p, miR-451a, regulate genes involved in bone remodeling	Prognosis	Discriminate patients with and without syndesmophytes, AUC of 0.82 sensitivity of 82% and specificity of 80% (11)

### Antibody-Based Diagnostic Biomarkers

#### Anti-CD74

Sera from patients with axSpA have been used to screen for autoantibody reactivity against new autoantigens in a protein array derived from fetal brain tissue (6). This identified reactivity to the extracellular portion of CD74, which is also known as HLA class II γ-chain or invariant chain. CD74 is involved in the assembly of human histocompatibility leukocyte antigen (HLA) class II molecules and its extracellular portion includes thyroglobulin type 1 and class II-associated invariant chain peptide (CLIP) domains. It may influence B cell differentiation and binds macrophage inhibitory factor leading to activation of nuclear factor κB and thereby expression of pro-inflammatory mediators (29).

A first ELISA that detected full-length human CD74 demonstrated reactivity in 56% of 41 patients with radiographic axSpA and 5% of 100 healthy blood donors (6). A second ELISA for IgG antibodies against the CLIP domain of the CD74 protein demonstrated reactivity in 69% of 216 patients with axSpA and in 97% of patients with a duration of inflammatory back pain of <1 year (6). Reactivity declined after the 1st year with reactivity of 78% at 11–20 years after onset and 65% after >20 years onset. Reactivity was observed in 45% of sera from 40 patients with psoriatic arthritis (PsA), 11% of 80 patients with rheumatoid arthritis (RA), 15% of 40 patients with system lupus, and only 1 of 125 (0.8%) blood donors. A second study reported anti-CLIP antibodies in 85.1% of 94 patients with axSpA vs. 7.8% of 51 controls (7). Sensitivity was 85.1% and specificity 92.2% while the positive LR for anti-CLIP antibodies to diagnose axSpA was 10.9 and the negative LR was 0.08.

These first two reports were based on sera that had been frozen for about 10 years. In subsequent development of the ELISA assay it became apparent that fresh sera contain soluble CD74, which interferes with binding of autoantibodies targeting CD74. Soluble CD74 may degrade when sera are kept frozen for at least a year. Consequently, subsequent studies tested an ELISA where whole CD74 was incorporated as an antigen to increase the number of epitopes available for antibody binding thereby increasing the sensitivity of the assay. In addition, both IgG and IgA anti-CD74 antibodies were tested. In the first study of 100 patients with non-radiographic axSpA and symptom duration of <2 years 47% had anti-IgA and 17% had anti-IgG antibodies to CD74 as compared to 4.7% of non-SpA back pain controls, respectively (8). In a second study of patients with early axSpA (*n* = 274) and with non-SpA chronic back pain (CBP) (*n* = 319), 46.4% of axSpA patients and 47.9% of CBP controls had IgG antibodies to CD74 while 54.7% of axSpA patients and 37% of CBP controls had IgA antibodies to CD74 (9). This resulted in a PPV of 58.8% and an NPV of 59.1% for IgA anti-CD74, which is of insufficient diagnostic value in patients with early axSpA.

#### Antibodies to Microbes and Quantitative Metagenomics

Antibodies to a variety of microbial components implicated in the pathogenesis of axSpA were described as potential diagnostic biomarkers over a decade ago but more recent research has focused on the gut microbiome and differences from healthy controls for potential diagnostic signatures. Quantitative metagenomics of gut microbial DNA from 211 Chinese individuals using deep shotgun sequencing demonstrated that 23,709 genes and 12 metagenomic species were differentially expressed between patients with axSpA and healthy controls (30). There was increased abundance of Prevotella species and decrease in Bacteroides species. Diagnostic algorithms that provided high discriminatory capacity between patients and controls [AUC of 90–95% in receiver operating curve analysis (ROC)] were derived using a subset of these gut microbial biomarkers. This work will require extensive replication studies to test generalizability to other patient populations.

#### Antibodies to Protein Phosphatase Magnesium-Dependent 1A (PPM1A)

A recent analysis assessed antibody reactivity in sera from individuals with pulmonary artery hypertension (*n* = 23), RA (*n* = 21), juvenile idiopathic arthritis (*n* = 15), psoriatic arthritis (PsA; *n* = 34), psoriasis (*n* = 6), and axSpA (*n* = 16) using high-density protein microarrays, containing 8,087 human proteins (10). Antibodies targeting protein phosphatase magnesium-dependent 1A (PPM1A), a Serine/Threonine protein phosphatase, were identified in patients with axSpA. This enzyme regulates bone morphogenetic protein (BMP) and Wingless (Wnt) signaling pathways and is a known inhibitor of transforming growth factor beta (TGF-β) signaling. Findings were independently confirmed in 45 Korean patients with axSpA, 20 patients with RA, and 30 healthy controls. Sensitivity and specificity were 66.7 and 73.3% for axSpA, respectively, when anti-PPM1A antibodies >2 SD above control were considered positive. Anti-PPM1A antibody levels were also higher in sera from rats transgenic for HLA-B27 and human β2-microglobulin although this was observed irrespective of clinically-evident arthritis. PPM1A was expressed in synovial tissue samples from patients with AS but no other diseases and overexpression in a pre-osteoblastic cell line increased alkaline phosphatase activity and nodule formation. Conversely, PPM1A knockdown significantly decreased expression of type I collagen and osteocalcin during differentiation. Levels of anti-PPM1A autoantibody were higher in patients with more extensive radiographic sacroiliitis. Moreover, levels decreased in patients treated with anti-tumor necrosis factor (anti-TNF) therapies, and this change was positively correlated with the change in disease activity. Despite these interesting links with the pathogenesis of disease, the performance of this assay is insufficient for diagnostic purposes.

### Candidate Diagnostic Biomarkers From Expression and Metabolomics Profiling

#### Gene Expression

A first meta-analysis of datasets based on publicly available gene expression arrays identified 905 differentially expressed genes in patients with axSpA compared to healthy controls, the most significant pathways being related to antigen processing and presentation (31). In one report, RNA sequence analysis revealed 19 serum microRNAs (miRNAs) that were differentially expressed in patients with axSpA compared to controls (32). MiRNAs are small non-coding RNAs (~22 nucleotides long), which modulate the expression of target genes at the post-transcriptional level, mostly reducing expression. Higher expression of miR-146a-5p, miR-125a-5p, miR-151a-3p, and miR-22-3p, and lower expression of miR-150-5p and miR-451a was observed in patients with axSpA when compared to healthy controls (11). The areas under the curve (AUC) for individual miRNAs ranged from 0.614 to 0.781 indicating low accuracy of these microRNAs as diagnostic biomarkers for axSpA while the AUC for, the six-miRNA composite signature reached 0.96. Pathway analysis revealed these miRNAs as targeting inflammatory genes. One differentially expressed miRNA, mi-R-10b, has been reported to decrease IL-17A production due to suppression of mitogen-activated protein kinase 7 (MAP3K7) in CD4+ T lymphocytes (33).

Recent expression studies have focused on diseased tissues and a combined analysis of all RNA moieties. A study of hip ligaments from patients with axSpA revealed differential expression of 574 mRNAs, 661 long non-coding RNAs, and 22 miRNAs in patients with axSpA compared to healthy controls (34). These RNAs are involved mainly in immune regulation, intercellular signaling, osteogenic differentiation, protein synthesis, and degradation. Two miRNAs, miR-17-5p and miR-27b-3p, were identified that could increase the differentiation pathways of fibroblasts toward osteogenesis. These candidate biomarkers require further validation in longitudinal studies of patients with unselected back pain.

#### Metabolomics

Several studies have assessed the metabolome using mass spectrometry to identify diagnostic profiles in serum, plasma, urine, and ligament tissues of hip joints from patients with axial SpA (35, 36). These have highlighted differences between AS patients and controls in metabolites related to intestinal microbial metabolism such as hippurate, glycine, tryptophan, phenylyacetylglycine, and butyrate. Differences in phospholipid and triglyceride metabolism included the metabolites acetoacetate, acetone, and 3-hydroxybutyrate and two branched amino acids leucine and valine. It is possible that combinations of metabolites could lead to a diagnostic profile that can be reproducibly validated in larger datasets, which is necessary to address the confounding effects of various comorbidities that often exist in patients with axSpA.

## Disease Activity Biomarkers

Assessment of disease activity in axSpA is constrained by the lack of physical findings indicative of inflammation, especially in early disease, and the lack of sensitivity of the most widely used laboratory marker, CRP. Moreover, the use of high sensitivity CRP or alternative acute phase reactants, such as serum amyloid A, does not enhance sensitivity over the established CRP assay. Major limitations of biomarker studies conducted to date include their cross-sectional design and validation with benchmarks such as the CRP and patient self-reported measures of symptoms, rather than objective measures such as MRI inflammation of the SIJ and spine, which has itself been validated with histopathological confirmation of inflammation. Identification of biomarkers that are more sensitive and responsive than CRP is a major unmet need in the field. Moreover, change in the biomarker should independently reflect change in the degree of MRI inflammation when adjusted for covariates.

### Biomarker Mediators of Inflammation

#### Calprotectin

The calcium-binding protein calprotectin is a heterodimer of S100A8 and S100A9 that has been examined in synovial tissue of patients with axSpA. It is expressed by macrophages and neutrophils in synovial tissue, in the colon of patients with inflammatory bowel disease (IBD), and in lesional skin of patients with psoriasis. Levels of calprotectin in feces reflect the degree of neutrophil inflammation in the gut and its measurement in feces has been helpful in discriminating IBD from irritable bowel syndrome. Fecal levels also correlate with clinical, endoscopic, and histologic parameters of disease severity in IBD but it may also be increased in enteropathy caused by nonsteroidal anti-inflammatory drugs (NSAIDs) (37–39). It is thought to mediate its pro-inflammatory effects through toll-like receptor 4 (TLR4).

In a study of 262 AS patients and 260 health controls, serum levels were higher in AS patients with AUC of 0.88 by ROC analysis and sensitivity and specificity of 80.2 and 92.7% at the optimal cut off level of 151.5 ng/mL (12). Weak correlation was observed with CRP, Bath AS Disease Activity Index (BASDAI), and serum levels of interleukin-1 beta (IL1β), IL17, and TNF-α at a cross-sectional level. In a study that evaluated fecal calprotectin, weak correlations were also reported between levels of fecal calprotectin and CRP, BASDAI, and the AS Disease Activity Score (ASDAS). This was observed both at baseline and after 5 years follow up in 204 patients with AS but correlations between changes in biomarker levels and disease activity parameters were not reported (13). Serum and fecal calprotectin and CRP were each independently associated with microscopic bowel inflammation in patients with early axSpA (14). Combined elevation of CRP and serum calprotectin was associated with a frequency of bowel inflammation of 64% compared to a frequency of only 25% in patients with low levels of both biomarkers. The assessment of fecal calprotectin provided further differentiation. In a further recent report, elevated fecal calprotectin (>100 μg/g) was significantly associated with Crohn's disease where this was evaluated using video capsule endoscopy (odds ratio 45) (15). The optimal cut-off value for fecal calprotectin for predicting Crohn's disease by capsule endoscopy was 132 μg/g (sensitivity = 66.7%, specificity = 76.9%, and AUC of 0.75). Further validation of this biomarker should incorporate assessment of MRI inflammation as a primary endpoint.

#### Cytokines

Interleukin 6 (IL6) has been tested for associations with MRI inflammation in patients with axSpA receiving the anti-TNF agent golimumab (16). Additional biomarkers that comprised the panel tested for associations with MRI inflammation included intracellular adhesion molecule-1, complement component 3 (C3), CRP, haptoglobin, leptin, tissue inhibitor of metalloproteinase (TIMP)-1, and serum amyloid-P. Although baseline levels of IL6 and the change in levels after institution of treatment correlated significantly with change in MRI inflammation it was not clear that this association occurred independently of CRP, which correlates with both IL6 and MRI inflammation. All other correlations tested between biomarkers in this panel and MRI inflammation in the spine yielded insignificant relationships. Two reports have recently described cross-sectional associations between hepatocyte growth factor (HGF) and acute phase reactants (17, 18). HGF is secreted by mesenchymal cells and has pleiotropic effects that include suppression of inflammation and enhanced osteoblastic differentiation of mesenchymal cells. Consistent associations between any cytokines and parameters of inflammation, especially MRI, have yet to emerge.

#### Tissue Turnover Biomarkers

Tissue remodeling is observed in inflammatory joint disease and is associated with degradation of extracellular matrix (ECM) proteins and intracellular proteins by proteases, which results in generation of a range of new protein fragments (neoepitopes). These processes provide opportunities to assess a variety of biomarkers that could be relevant to disease activity such as metalloproteinases and antibodies to *de novo* protein neoepitopes Older reports had described weak associations between metalloproteinases and disease activity measures, though not MRI inflammation. Serological quantification of collagen degradation fragments of types I (C1M), II (C2M), IV (C4M), V (C5M), and VI (C6M) collagens generated by matrix metalloproteinase (MMP) has demonstrated excess tissue remodeling in patients with axSpA compared to controls. Vimentin is a type III intermediate filament protein that is also degraded by MMP and one fragment then undergoes citrullination (VICM). Highly significant correlation was recently reported between C1M, C6M, and VICM with CRP although correlations with MRI inflammation were not assessed (19). This data is consistent with an older report (40) and suggests that further longitudinal studies of these biomarkers are warranted. No additional biomarkers reflecting tissue turnover of bone, cartilage, or connective tissue have emerged as having major associations with disease activity in axSpA.

### Candidate Disease Activity Biomarkers From Expression Profiling

The miRNAs, miR-146a and miR-155, are expressed in patients with axSpA and the level of expression correlates with parameters of disease activity such as the CRP and the BASDAI (20, 32). miR-146a regulates innate immune responses through intervening in toll-like receptor pathways and is one of the miRNAs comprising a diagnostic signature (miR-146a−5p, miR-125a-5p, miR-151a-3p, miR-22–3p, and miR-451a) shown to be associated with plasma levels of CRP and TNF-alpha (11). In addition to elevated expression levels of these miRNAs in patients with active vs. non-active disease, lymphocytes from patients transfected with mimics of miR-125a-5p, miR-451a, and miR-22–3p had reduced expression of pro-inflammatory cytokines TNF-α, IL-1β, and IL-5. These promising novel biomarkers should undergo further validation studies.

## Theragnostic Biomarkers

CRP is still the only biomarker that is currently used in clinical practice to help select patients for treatment and together with MRI inflammation has been shown to predict treatment response to a TNFi. The change in serum calprotectin in the 1st month of treatment may also help to predict treatment response to a TNFi agent although predictive capacity for major treatment response was limited (AUC 0.69) (12).

A high-dimensional, multi-parametric flow cytometric analysis was applied to identify cell surface biomarkers as potential immunophenotypic predictors of response to anti-TNF agents in 31 patients with axSpA (41). Low frequency of a subpopulation of NK cells expressing CD8 was associated with a lack of response (AUC 0.79). This was primarily observed with one specific anti-TNF agent, namely, etanercept. This proof of concept study will require further validation in larger cohorts.

## Prognostic Biomarkers

Development of ankylosis is still primarily assessed using plain radiography, especially of the spine. There are major limitations to this endpoint because reliable detection of progression requires a 2 years' time frame and it demonstrates limited sensitivity to change. Consequently, the Outcome Measures in Rheumatology (OMERACT) Soluble Biomarker International Working Group Biomarker has recommended that validation of prognostic biomarkers in axSpA should focus on demonstrating that short term change in the biomarker after instituting treatment is predictive of long-term change in structural progression independently of any other predictors (5). Moreover, the biomarker should enhance the predictive capacity of prediction models based on clinical and lab measures currently used in clinical practice. C-telopeptide of type I collagen (CTX-I) is an example of an appropriately validated prognostic biomarker because of its ability to predict risk for fracture. If CTX-I decreases after treatment with an antiosteoporotic agent, this will predict long-term change both in bone mineral density (BMD) and in fracture risk. Moreover, CTX-I enhances the predictive capacity of models that include other predictors such as BMD, age, and prior fracture (42, 43).

### Biomarkers Related to Inflammation

Relatively few studies conducted in axSpA have been prospective in study design. These showed that CRP was consistently associated with progression while limited data from isolated reports also implicated additional inflammatory biomarkers such as serum IL6 and calprotectin (16, 21–26). Data supporting a prognostic role for vasoactive endothelial growth factor could not be replicated (44, 45).

Serum levels of macrophage migration inhibitory factor (MIF) have been reported to be elevated in axSpA, especially in those with disease progression on follow up and in those with existing syndesmophytes at baseline (27). Higher levels of MIF were also observed in synovial fluid from peripheral large joints and ileal tissue of patients with AS. MIF induced mineralization of primary osteoblasts in a dose-dependent manner, upregulated genes involved in osteogenesis and triggered stabilization of a known mediator of osteoblastic activity, β-catenin, suggesting a direct role for MIF in enhancing mineralization by osteoblasts. MIF has previously been reported to be elevated in patients with psoriasis, uveitis, and inflammatory bowel disease. It was postulated that bacteria could trigger ileal production of MIF, especially in CD68+ macrophages and Paneth cells, which in turn activates CD14+ monocytes to produce TNF and primes immune cells to express IL17.

### Cytokines

Serum levels of Th1, Th2, and Th17 cytokines were assessed at baseline in 443 patients from a French cohort of patients with early axSpA and 79 controls using a Luminex ELISA platform that assessed 17 cytokines (IL-1β, IL-4, IL-6, IL-10, IL-17A, IL-17F, IL-21, IL-22, IL-23, IL-25, IL-31, IL-33, IFNγ, soluble CD40 ligand (sCD40L), and TNFα) (46). Only levels of IL31 and sCD40L were increased in patients. Elevated IL-31 was found in 61% of patients vs. 18% of controls and was significantly associated with less structural damage. IL31 binds to a heterodimeric receptor, which comprises the IL-31 receptor A (IL-31RA) and the Oncostatin M receptor (OSMR) and mediates signaling though the following pathways: Jak/STAT, PI3K/AKT, MAPK. It is found in intestinal mucosa from patients with colitis and aqueous humor from patients with acute anterior uveitis (47, 48). Levels correlate with those of DKK-1, IL6, and TNFα and are increased in post-menopausal osteoporosis (46, 49).

### Tissue Turnover Biomarkers

A recent study of biomarkers of tissue turnover from sera of a prospective cohort did not confirm an earlier observation that citrullinated metalloproteinase degraded fragments of vimentin (VIMC) were independently predictive of progression (19, 40). This study raised the possibility that these protein fragments could be destroyed by long-term storage at −80C for up to 15 years because baseline VICM levels were much lower in this cohort. Levels of another biomarker fragment, C1M, were also lower than had been reported previously in patients with axSpA.

### Biomarkers Regulating Bone Formation

DKK-1 and sclerostin are mediators that inhibit signaling through the Wnt pathway, which is vital to bone formation. Older studies had independently reported lower levels of DKK-1 and sclerostin in axSpA compared to healthy controls suggesting that they could be biomarkers for structural progression. However, other studies have reported normal or high levels. Similar inconsistencies have been reported for measurement of serum Wnt-3a levels. There have been no recent studies that have attempted to clarify these discrepancies.

### Adipokines

The adipokine, visfatin, has direct activating effects on osteoblasts and has been shown in one study to predict radiographic progression in axSpA independently of CRP (28). Low serum levels of leptin and high molecular weight form of adiponectin were recently reported to be associated with radiographic progression in males independently of CRP and the baseline level of structural damage (50). Male gender is a significant confounder in studies evaluating adipokines because levels are lower in males.

## Candidate Prognostic Biomarkers From Expression Profiling

Isobaric tags for relative and absolute quantitation (ITRAQ) is a methodology that is increasingly being used for generating quantitative proteomic expression profiles in many autoimmune diseases. A pilot proteomic analysis of peripheral blood mononuclear cells (PBMCs) analyzing 1,232 proteins using iTRAQ showed differential expression of 183 proteins between patients with axSpA and healthy controls (51). Four of these proteins include cathepsin G (CTSG), neutrophil defensin 3 (DEFA3), protein tyrosine phosphatase receptor type C (PTPRC), and peroxiredoxin-1(PRDX1). Each of these proteins is involved in the process of cell death.

MiRNA-199a-5p is a recently discovered miRNA that regulates autophagy in T cells. Its expression was decreased in T cells of AS patients and its transfection into T cell lines was associated with suppression of proinflammatory cytokines (52). Its expression in T cells from AS patients correlated negatively with structural damage assessed by radiography. Gene expression array analysis of hip joint ligaments has demonstrated upregulation of miR-17-5p, which regulates several mediators such as BMP-2, SMAD5, and SMAD7, involved in osteogenesis (53). Expression of several BMPs correlates with radiographic severity of AS and is upregulated by proinflammatory cytokines. Moreover, suppression of BMP expression by silencing MMP2 inhibits osteogenesis of fibroblasts in AS (54).

From a six-plasma microRNA signature shown to have diagnostic potential for AS, a signature of four mi-RNAs (miR-125a-5p, miR-151a-3p, miR-150–5p, and miR-451a) were shown to discriminate AS patients with and without syndesmophytes with an AUC of 0.82, sensitivity of 82.4% and specificity of 80% (11). These miRNAs regulate expression of a number of genes directly related to both inflammation and bone remodeling in axSpA, such as AKT serine/threonine kinase 3 (AKT3), fibroblast growth factor receptor 1 (FGFR1), integrin subunit beta 3 (ITGB3), SMAD family member 4 (SMAD4), and tumor protein P53 (TP53).

## Conclusions and Future Directions

The primary advance in biomarker research of axSpA over recent years is the recognition that fecal calprotectin appears to be a useful indicator of bowel inflammation in patients with axSpA. Increasing evidence supports the use of this tool in the assessment of axSpA patients with symptoms suggestive of bowel inflammation leading to expedited referral to a gastroenterologist if the test is positive. Conversely a normal test has high NPV for excluding inflammatory bowel disease. Although additional biomarkers of potential value to axSpA have been identified in discovery studies most have not been replicated and/or assessed in prospective studies with appropriate outcomes as gold standard for the presence of disease and its severity.

High-throughput detection and quantification of serum proteins for biomarker discovery has been limited by the low sensitivity of proteomic profiling technologies such as 2D gels and mass spectrometry. Antibody-based multiplexing platforms have increased sensitivity but such assays cannot be significantly multiplexed because cross-reactivity of secondary antibodies to surface-immobilized proteins reduces specificity. A new method for proteomic analysis includes the use of Slow Off-rate Modified Aptamer (SOMA) reagents. These consist of a short single-stranded DNA sequence incorporating a series of modifications that give the SOMAmer “protein-like” appendages that allow the SOMAmer to tightly bind its target protein (55). In a recent prospective study of 5,457 individuals older than 65 years of age participating in the AGES Reykjavik study, an updated array of 5,034 SOMAmers that recognized 4,137 individual human proteins was used to study the serum proteome (56). The human serum proteome appeared as 27 functionally distinct network modules of proteins, ranging in size between 20 and 921 proteins, produced by many tissues of the body. They are associated with cardiovascular and metabolic diseases, as well as overall survival. Subsequent steps in the identification of biomarkers for axSpA are likely to explore the use of such technology in carefully phenotyped patient populations, especially those with prospective follow up and objective measures of the presence of disease and its severity.

## Author Contributions

The author confirms being the sole contributor of this work and has approved it for publication.

### Conflict of Interest Statement

WM is co-inventor of the 14-3-3η biomarker technology platform and has received consulting fees from Augurex Life Sciences Corporation, and milestone and royalty payments for the 14-3-3η technology.
